# *Cudrania cochinchinensis* attenuates amyloid β protein-mediated microglial activation and promotes glia-related clearance of amyloid β protein

**DOI:** 10.1186/1423-0127-20-55

**Published:** 2013-08-02

**Authors:** Chung-Jen Wang, Chien-Chih Chen, Huey-Jen Tsay, Feng-Yi Chiang, Mine-Fong Wu, Young-Ji Shiao

**Affiliations:** 1 Division of Geriatrics, Cheng Hsin General Hospital, Taipei, Taiwan; 2 Department of Biotechnology, HunKuang University, Taichung 433, Taiwan; 3 Institute of Neuroscience and Brain Research Center, National Yang-Ming University, Taipei 112, Taiwan; 4 Institute of Biopharmaceutical Science, National Yang-Ming University, Taipei 112, Taiwan; 5 Division of Basic Chinese Medicine, National Research Institute of Chinese Medicine, Taipei 112, Taiwan

**Keywords:** Neuroinflammation, *Cudrania cochinchinensis*, Microglia, Mixed glial culture, Amyloid β, Alzheimer’s disease

## Abstract

**Background:**

Microglial inflammation may significantly contribute to the pathology of Alzheimer’s disease. To examine the potential of *Cudrania cochinchinensis* to ameliorate amyloid β protein (Aβ)-induced microglia activation, BV-2 microglial cell line, and the ramified microglia in the primary glial mixed cultured were employed.

**Results:**

Lipopolysaccharide (LPS), Interferon-γ (IFN-γ), fibrillary Aβ (fAβ), or oligomeric Aβ (oAβ) were used to activate microglia. LPS and IFN-γ, but not Aβs, activated BV-2 cells to produce nitric oxide through an increase in inducible nitric oxide synthase (iNOS) expression without significant effects on cell viability of microglia. fAβ, but not oAβ, enhanced the IFN-γ-stimulated nitric oxide production and iNOS expression.

The ethanol/water extracts of *Cudrania cochinchinensis* (CC-EW) and the purified isolated components (i.e. CCA to CCF) effectively reduced the nitric oxide production and iNOS expression stimulated by IFN-γ combined with fAβ. On the other hand, oAβ effectively activated the ramified microglia in mixed glial culture by observing the morphological alteration of the microglia from ramified to amoeboid. CC-EW and CCB effectively prohibit the Aβ-mediated morphological change of microglia. Furthermore, CC-EW and CCB effectively decreased Aβ deposition and remained Aβ in the conditioned medium suggesting the effect of CC-EW and CCB on promoting Aβ clearance. Results are expressed as mean ± S.D. and were analyzed by ANOVA with post-hoc multiple comparisons with a Bonferroni test.

**Conclusions:**

The components of *Cudrania cochinchinensis* including CC-EW and CCB are potential for novel therapeutic intervention for Alzheimer’s disease.

## Background

*Cudrania cochinchinensis* is a Chinese folk medicine with anti-inflammation, anti-microbial, and antioxidant properties [1]. A xanthone derivative isolated from *C. cochinchinesis* has been found to inhibit lipopolysaccharide (LPS)-induced nitric oxide production in RAW264.7 macrophages [2]. However, the therapeutic potential of *C. cochinchinesis* on central nervous system (CNS)-related diseases has yet to be studied.

Microglia, the brain-resident macrophages, exhibits several morphological features that distinguish them from peripheral macrophages. These include ramified branches in the steady-state phenotype and the amoeboid morphology of renewed and activated phenotypes [3,4]. Steady-state microglia exhibits a resting-like phenotype, characterized morphologically by extensively ramified processes that continuously monitor its surroundings in the CNS [5]. The property of the activated amoeboid microglia is similar to macrophages. However, the primary determinants of ramified phenotype and amoeboid phenotype of microglia under pathological conditions in the CNS are less well defined and may be different from those of macrophages in the peripheral tissues.

In neurodegenerative diseases, the number of activated microglia increase was observed near the foci of lesions [6,7]. The priming of microglia responding to stimuli depends on a set of growth factors, such as macrophage colony-stimulating factor (M-CSF) and IFN-γ [8,9]. Microglia may exert protective and pathogenic functions in the CNS [9,10] and may exert beneficial and detrimental effects on AD therapy. AD pathology is characterized by the accumulation of Aβ containing neuritic plaques [11]. In addition to direct neurotoxicity, oligomeric Aβ (oAβ) and fibrillary Aβ (fAβ) are known to activate microglia and produce pro-inflammatory mediators; the neurotoxicity may impede the treatment of AD [10]. On the contrary, microglia may protect neurons through the production neurotrophic factors [12], clearing Aβ by phagocytosis [13,14], and degrading Aβ by the secreted enzymes such as insulin degrading enzyme (IDE) [15]. Therefore, it is essential to find an appropriate therapeutic intervention, especially for herbal medicines, aimed at modulating microglia activities from a reduction of the detrimental effects and promotion of a protective role.

We use BV-2 microglial cell line and the primary ramified microglia in the mixed glial culture as an experimental platform. We study the effects of *C. cochinchinensis* on the therapeutic potential of Alzheimer’s disease (AD). The effects on amyloid β protein (Aβ)-mediated nitric oxide production and inducible nitric oxide synthase (iNOS) expression in the interferon-γ (IFN-γ) primed BV-2 cells were studied. On the other hand, the effect of these components on Aβ-induced morphological change of steady state ramified microglia in the mixed glial culture and Aβ clearance by microglia were investigated. We found that CC-EW and CCB reduced the nitric oxide production and iNOS expression mediated by fAβ plus IFN-γ. Furthermore, the recoveries of the Aβ-mediated morphological change of primary microglia and the promotion of microglial clearance of Aβ confirmed the effects of CC-EW and CCB on the ramified microglia.

## Methods

### Reagents

Medium for cell culture was purchased from Invitrogen (Carlsbad, CA, USA). Mouse anti-actin antibody and synthetic Aβ1-42 were purchased from Millipore (Billerica, MA, USA). Anti-Aβ1-17 antibody (clone 6E10) was from Signet (Dedham, MA). Anti-Aβ17-24 antibody (clone 4GB) was from Oncogen (San Diego, CA, USA). Anti-Iba-1 antibody was from Abcam (Cambridge, CB4 0WN, NK). Anti-iNOS antibody was from Santa Cruz (Santa Cruz, California, USA). Enhanced chemiluminescence detection reagents, anti-rabbit and anti-mouse IgG antibody conjugated with horseradish peroxidase were obtained from GE Healthcare (Buckinghamshire, UK). All other reagents were purchased from Sigma (St. Louis, MO, USA) or Merck (Darmstadt, Germany).

### Plant material, extraction, and isolation

**Figure 1:**
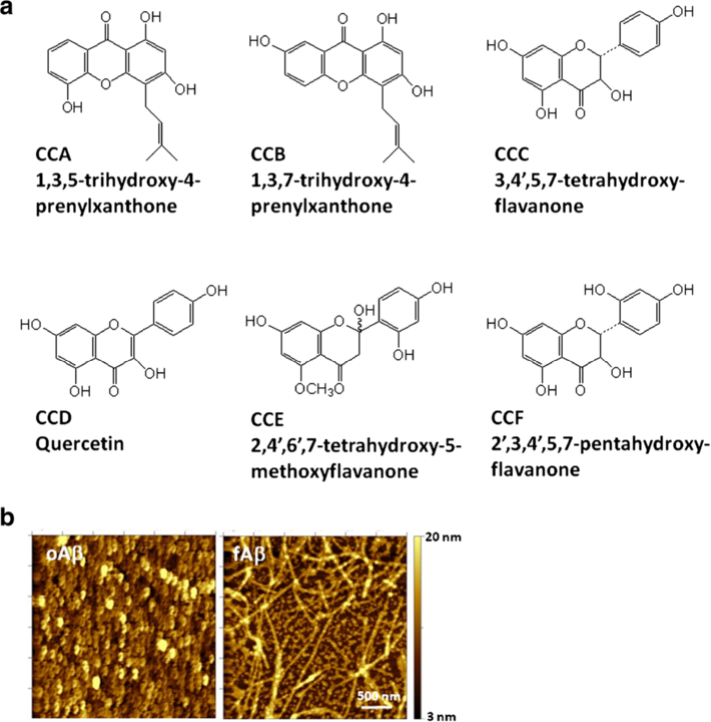
**The structure of the compounds and the amyloid peptides used in this study. (a)** The structure and chemical name of the compounds isolated from *C. cochinchinesis*. **(b)** The representing images of the conformation of the prepared Aβ aggregates.

The extraction of *C. cochinchinensis* were processed according to the method previously described [2]. Briefly, the air-dried roots of *C. cochinchinensis* were extracted four times with methanol (MeOH) under reflux to give the MeOH extract. The MeOH extract was partitioned with an ethyl acetate (EtOAc) and water mixture (1:1) to give the EtOAc extract and the water extract (CC-EW). The EtOAc extract was subjected to silica gel column chromatography eluting with n-hexane: EtOAc/MeOH to give eight fractions. Fractions was further separated by HPLC (Cosmosil 5C18-AR, Nacalai Tesque, Tokyo, Janpan) to obtain CCA to CCF. The structure of each compound (Figure 1a) was elucidated with mass and NMR spectroscopy in comparison to the published data. The purity of each compound was >98% as judged by HPLC and ^1^H NMR.

### Cell culture

BV-2 cells were cultured in Dulbecco’s Modified Eagle’s Medium (DMEM) containing 10% fetal bovine serum (FBS). For treatment, the cell medium was replaced with DMEM containing 1% FBS. Primary cultures of neonatal cortical microglia were prepared from the cerebral cortex of Sprague–Dawley rat pups at postnatal day 5 [16]. Briefly, neonatal day 5 pups were anaesthetized with ether and sacrificed by decapitation. The Institutional Animal Care and Use Committee at the National Research Institute of Chinese Medicine has approved the animal protocol used in our study (IACUC number: 95-P-08 and P-99-18). Primary mixed glial cells were prepared from the cerebral cortex and maintained in DMEM/F12 medium containing 10% FBS for 3 days. The medium was changed with fresh culture medium and incubated for 2 days. Thereafter, the cells were cultured in Neurobasal medium/B27 supplement for 1 to 10 days.

### Preparation and biochemical characterization of oAβ and fAβ

oAβ and fAβ were prepared as described [17,18]. A diluted solution of Aβs was spotted onto a mica slide and scanned using an Agilent® 5400 atomic force microscope (Molecular Imaging Corporation, Tempe, AZ) as described previously [19]. Figure 1(b) shows the morphology of the prepared oAβ and fAβ.

### Measurement of nitrite

Nitrite content (nitric oxide release) was measured by incubating culture medium with an equal volume of Griess reagent (0.05% N-(1-naphthyl)-ethylene-diamine dihydrochloride, 0.5% sulfanilamide, and 1.25% phosphoric acid). After incubation, the optical density was detected at a wavelength of 540 nm using a microplate reader with NaNO_2_ as standard.

### MTT assay

The reduction of 3-[4,5-dimethylthiazol-2-yl]-2,5-diphenyl- tetrazolium bromide (MTT) was used to evaluate cell viability. Cells were incubated with 0.5 mg/ml MTT for 1 h. The formazan particles were dissolved with DMSO. OD_600 nm_ was measured using an ELISA reader.

### Immunoblotting

After treatment, culture media were collected and cells were washed with ice-cold PBS three times. Cells were harvested in lysis buffer (50 mM Hepes pH7.5, 2.5 mM EDTA, 1 mM phenylmethylsulfonyl fluoride, 5 μg/ml aprotinin, and 10 μg/ml leupeptin) and cell lysates were prepared. Equal protein amounts of cell lysate and an equal volume of culture medium were subjected to SDS-polyacrylamide gel electrophoresis and immunoblotting. Fujifilm LAS-3000 (Tokyo, Japan) was used to detect and quantify the immunoreactive protein.

### Immunocytochemistry

Treated cells were fixed with 4% paraformaldehyde (in PBS) at room temperature for 15 min and permeabilized with 0.5% Triton X-100 (in PBS) for 10 min. Cells were blocked with 10% normal donkey serum (in PBS containing 0.5% bovine serum albumin, BSA) at room temperature for 2 h. Cells were treated to detect microglia and its activation using goat anti-Iba-1 and mouse anti-iNOS, respectively. Donkey anti-rabbit IgG or anti-goat IgG conjugated with cy5 and donkey anti-mouse IgG conjugated with fluorescein were used as secondary antibodies. For the study of Aβ deposition, anti-Aβ monoclonal antibody 4G8 and donkey anti-mouse IgG antibody conjugated with fluorescein were used as first and secondary antibodies, respectively.

### Morphological quantification

Metamorph (software) was employed to calculate cell area and convex area of microglia. Form factor was calculated by dividing cell area with convex area, which was described before [20].

### Quantification of Aβ1-42 in cells and culture medium

After treatment, culture media and cells were collected separately and subjected to determine the levels of Aβ1-42 using assay kits (Invitrogen, KHB3442). The detailed experiments were performed according to the manufacturer’s protocol. The conditioned medium was added to assay wells pre-coated with anti-amyloid antibody and incubated with detection antibody at room temperature for 3 h. After wash, the wells were incubated with hourse radish peroxidase anti-rabbit antibody for 30 min at romm temperature. The wells were then incubated with stabilized chromogen for 30 min at romm temperature. After adding stop solution, the wells were read at 450 nm.

### Statistical analysis

Results are expressed as mean ± S.D. and were analyzed by ANOVA with post-hoc multiple comparisons with a Bonferroni test.

## Results

### The nitric oxide production induced by LPS, IFN-γ, and IFN-γ combined with fAβ in BV-2 cells culture

**Figure 2:**
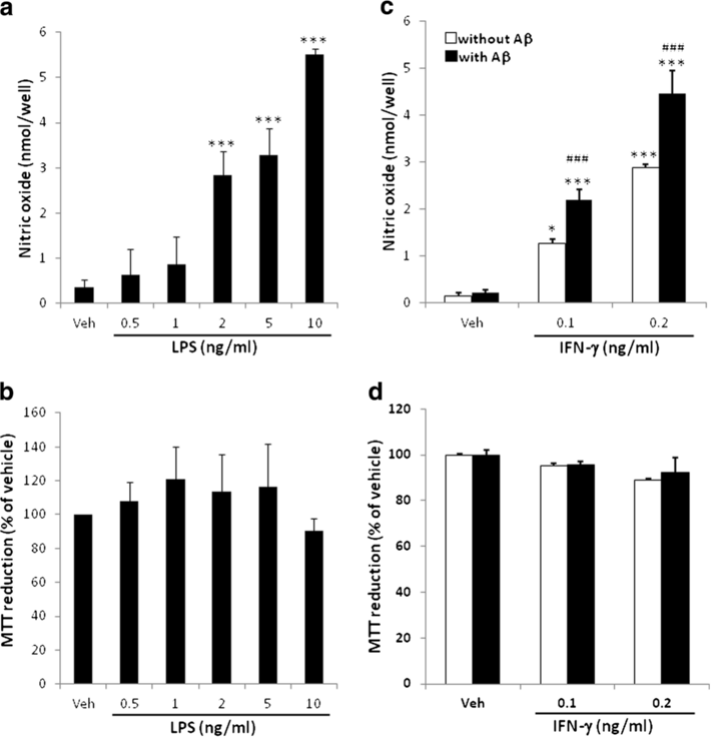
**Effect of LPS, IFN-γ, and IFN-γ + fAβ on nitric oxide production in BV-2 cells.** BV-2 cells were treated with indicated concentrations of LPS **(a, b)** or vehicle, 0.1 and 0.2 ng/ml IFN-γ alone, vehicle or FN-γ + 1 μM fAβ **(c, d)**, for 24 h. **(a, c)** After treatment, cultured medium was collected and the nitrite content was determined. **(b, d)** The cell viability of the treated cells was assayed by MTT reduction. Results are means ± S.D. from three independent experiments. Significant differences between cells treated with vehicle (Veh) and LPS or IFN-γ is indicated by *, p < 0.05, ***, p < 0.001. Significant differences between the cells treated with IFN-γ alone and IFN-γ combined with fAβ are indicated by ###, p < 0.001.

To determine the inflammatory potential of BV-2 cells, nitric oxide production was assayed after BV-2 cells were activated by different stimuli. LPS at 2 and 10 ng/ml stimulate BV-2 to produce 2.83 ± 0.54 and 5.52 ± 0.12 nmol/well nitric oxide, respectively, without significantly affect the cell viability (Figure 2a, b). IFN-γ at 0.1 and 0.2 ng/ml stimulates BV-2 to produce 1.27 ± 0.10 and 2.88 ± 0.07 nmol/well nitric oxide, respectively. As combined with 1 μM fAβ, the production of 0.1 and 0.2 ng/ml IFN-γ-stimulated nitric oxide productions were increased to 2.20 ± 0.22 and 4.46 ± 0.49 nmol/well, respectively (Figure 2c). The cell viability is also not affected by IFN-γ (Figure 2d). However, oAβ failed to enhance the IFN-γ-stimulated production of nitric oxide (data not shown).

### The effects of CC-EW and the compounds, CCA to CCF, on nitric oxide production induced by IFN-γ alone or combined with fAβ in BV-2 cells culture

**Figure 3:**
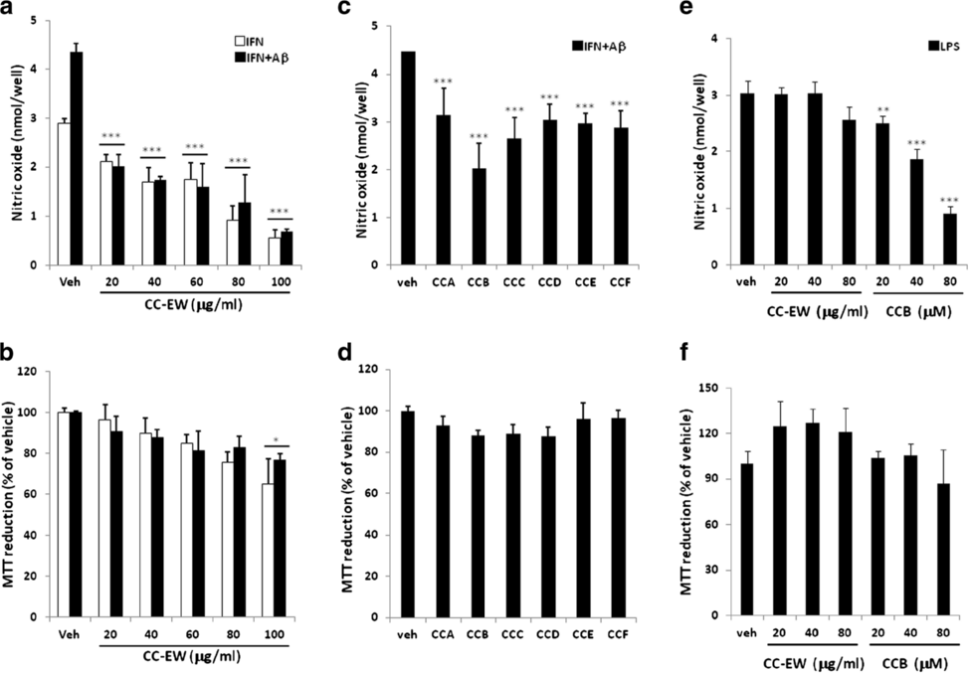
**Effect of *Cudrania cochinchinensis *on nitric oxide production induced by IFN-γ, fAβ and LPS in BV-2 cells.** BV-2 cells were treated with indicated concentration of CC-EW **(a, b, e, f)**, 20 μM of CCA to CCF **(c, d)** or indicated concentration of CCB for 2 h, and then treated with 0.2 ng/ml IFN-γ alone, IFN-γ combined with 1 μM fAβ or 10 ng/ml LPS for 24 h. **(a, c, e)** After treatment, the cultured medium was collected and the nitrite content was determined. **(b, d, f)** The cell viability of the treated cells was assayed by MTT reduction. Results are means ± S.D. from three independent experiments. Significant differences between the cells treated with vehicle (Veh) and CC-EW or the compound, CCA to CCF, are indicated by ***, p < 0.001.

To determine the anti-inflammatory effects of CC-EW, IFN-γ (0.2 ng/ml) alone or combined with fAβ (1 μM) were used as stimuli. CC-EW (20 μg/ml) reduced the nitric oxide production mediated by IFN-γ alone and combined with fAβ to 26.95 ± 4.79% and 53.79 ± 7.68% of the vehicle treated control cells, respectively (Figure 3a). The IC_50_ of CC-EW on the cells stimulated with IFN-γ alone and combined with fAβ was 58.28 ± 6.72 and 12.83 ± 3.36 μg/ml, respectively (Table 1), to suggest that CC-EW possess a higher inhibitory activity specifically to the stimulation by IFN-γ combined with fAβ than that by IFN-γ alone. On the contrary, CC-EW did not inhibit the stimulation by LPS (10 μg/ml) (Figure 3e). On the other hand, CC-EW in the concentration below 100 μg/ml did not significantly affect the viability of BV-2 cells (Figure 3d, f).

**Table 1:** **The IC**_**50 **_**of CC-EW and CCB for Aβ-mediated changes on microglia**

**Stimuli**	**Component**	**IC**_**50 **_**(μg/ml or μM)**^**a**^			
		**Nitric oxide**	**iNOS expression**	**Form factor**	**Aβ deposition**
IFN-γ	CC-EW	58.28 ± 6.73	nd	nd	nd
IFN-γ + Aβ	CC-EW	12.83 ± 3.36	40.83 ± 5.66	30.00 ± 3.65	32.67 ± 2.69
	CCB	11.01 ± 1.02	11.37 ± 1.63	10.02 ± 2.11	17.82 ± 1.78

a. The unit for IC^50^ is μg/ml for CC-EW and μM for CCB.

nd means not determined.

Six pure compounds isolated from CC-EW were also subjected to the inhibition assay of nitric oxide production stimulated by IFN-γ plus fAβ. The results showed that 20 μM of CCA, CCB, CCC, CCD, CCE, and CCF inhibit nitric oxide production to 66.67 ± 17.54, 46.67 ± 6.67, 57,78 ± 4.44, 66.67 ± 5.76, 66.67 ± 8.07, and 64.64 ± 6.67% of the vehicle treated control cells, respectively (Figure 3b). The results indicated that CCB is the most effective compounds and its IC_50_ is calculated as 11.01 ± 1.02 μM (Table 1). CCB also possesses inhibitory activity to stimulation by LPS (Figure 3e). All six compounds did not significantly affect the viability of BV-2 cells (Figure 3d, f).

### The effects of CC-EW and CCB on the iNOS expression induced by IFN-γ alone or combined with fAβ in BV-2 cells culture

**Figure 4:**
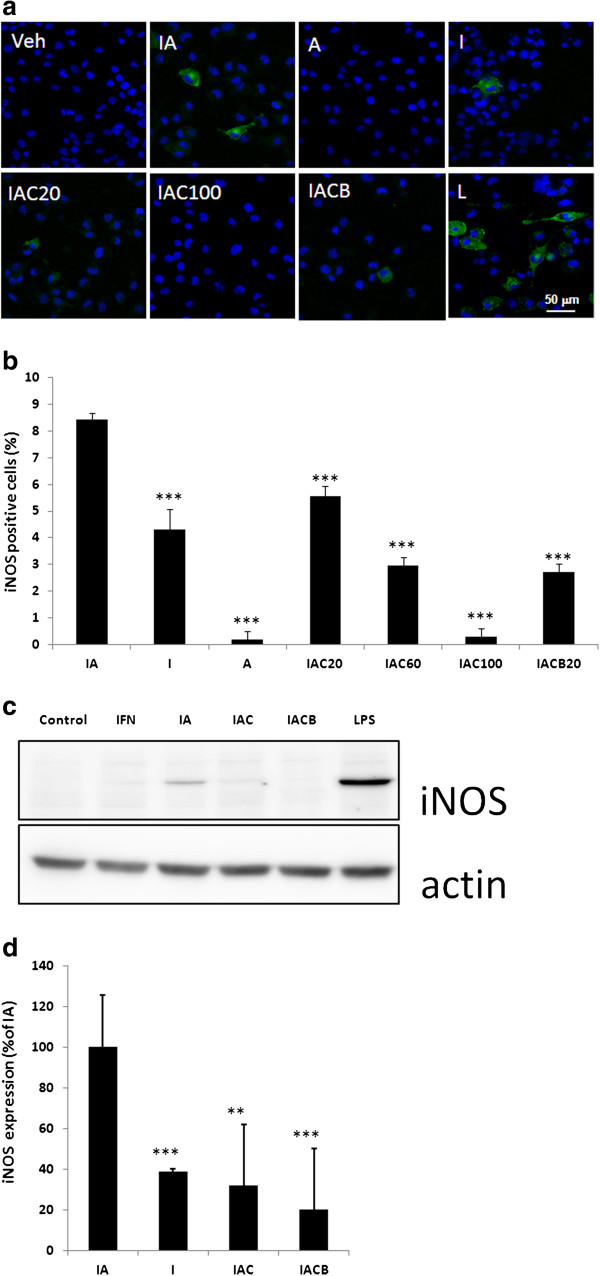
**Effect of *Cudrania cochinchinensis *on iNOS expression induced by IFN-γ combined with fAβ in BV-2 cells.** BV-2 cell were treated with 0.2 ng/ml IFN-γ (I), 1 μM fAβ (A), IFN-γ combined with fAβ (IA). Alternatively, BV-2 cell were treated with 20 μg/ml CC-EW (IAC) or 20 μM CCB (IACB) for 2 h, and then were treated with IFN-γ combined with fAβ for 24 h. Cell treated with 10 ng/ml LPS (L) was used as positive control. **(a)** Cells were fixed, subjected to iNOS staining by anti-iNOS antibody (green) and nucleus staining using Hoechst 33258 (blue). **(b)** The levels of iNOS positive cells (%) are total count from three different fields. **(c)** The level of iNOS in cell lysate was determined by immunoblotting using β-actin as internal standard. **(d)** The relative level of iNOS in cell lysate exhibited as percentage of that in IA. Results are means ± S.D. from three independent experiments. Significant differences between the IA and the other treatments are indicated by ***, p < 0.001.

Nitric oxide is produced by the activity of iNOS, which is expressed in activated inflammatory cells. To determine whether the modulation of nitric oxide production was attributable to the level of iNOS expression, the immunocytochemistry using anti-iNOS antibody was performed to detect iNOS expression in the treated BV-2 cells. IFN-γ significantly increases the percentage of iNOS-positive BV-2 cells (Figure 4a, b). fAβ did not increase the percentage of iNOS-positive BV-2 cells. However, it significantly enhances IFN-γ-induced iNOS expression. Alternatively, the positive control, LPS, extensively increase the percentage of iNOS-positive BV-2 cells. Use of IFN-γ combined with fAβ as stimuli, CC-EW and CCB significantly inhibits iNOS expression with IC_50_ at 40.83 ± 5.66 μg/ml and 11.37 ± 1.63 μM, respectively (Figure 4; Table 1). The results indicated that CCB is better than CC-EW on inhibiting the iNOS expression. The iNOS level was also determined from immunoblotting. The results show that IFN-γ combined with fAβ induced a significantly higher amount of iNOS expression than that induced by IFN-γ alone. Although the level is not as high as that stimulated by the positive control LPS. CC-EW reduced the iNOS expression induced by IFN-γ combined with fAβ to 35.24 ± 0.05% of that induced by IFN-γ plus fAβ. The results indicated that CC-EW significantly reduced the iNOS expression induced by IFN-γ combined with fAβ.

### The effects of LPS, IFN-γ, and oAβ on morphological change of the ramified microglia in mixed glial culture

**Figure 5:**
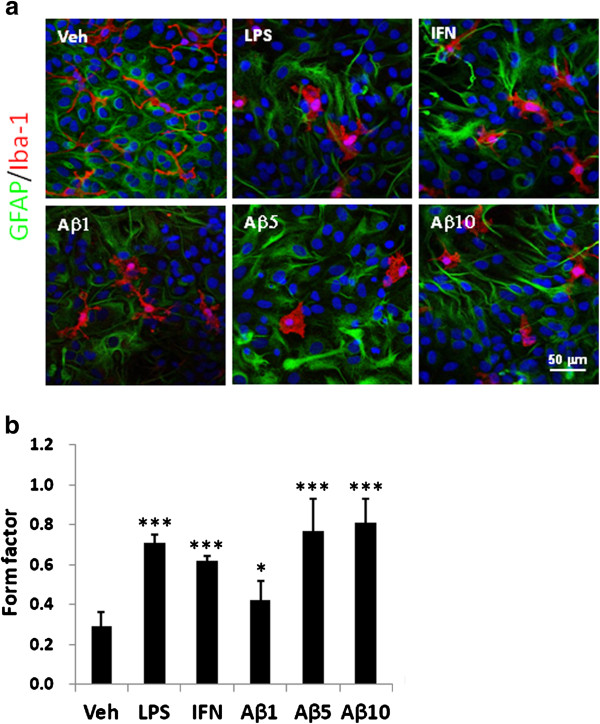
**Morphological alteration of the microglia stimulated by LPS, IFN-γ, oAβ alone or combined with IFN-γ.** Glial cells treated with vehicle (Veh), 10 ng/ml LPS, 0.5 ng/ml IFN-γ (IFN), or 1–10 μM oAβ (Aβ1, Aβ5, Aβ10) for 24 h. **(a)** Cells were fixed and subjected to immuno-staining of microglia by anti-Iba1 antibody (red), astrocyte by anti-GFAP antibody (green) and nuclei staining using Hoechst 33258 (blue). Results are repeated for three times, and represent photographs are shown. **(b)** Form factor of microglial morphology were calculated as describe in the Method section. Results are means ± S.D. from three independent experiments and total nine cells of each group were analyzed. Significant differences between the cells treated with vehicle and the other treatments are indicated by *, p < 0.05, ***, p < 0.001.

The morphological change of the ramified microglia in the primary mixed glial culture was further employed to examine the effect of Aβ on microglia activation. The microglia co-cultured with astrocyte displayed ramified processes with a small cell body and several long processes. The ramified morphology was altered to amoeboid as the cell was activated by 10 ng/ml LPS, 0.5 ng/ml IFN-γ (IFN), or 1 to 10 μM oAβ (Figure 5a). To quantify the morphological alteration of microglia, the form factor of single cell was calculated as described in the Method section. The results showed that LPS and IFN-γ significantly increased the form factor of microglia from 0.29 ± 0.07 to 0.71 ± 0.04 and 0.62 ± 0.02, respectively (Figure 5b). oAβ, at 1 and 5 μM, increase the form factor of microglia from 0.29 ± 0.07 to 0.42 ± 0.10 and 0.77 ± 0.16, respectively. On the other hand, the morphology of astrocyte was altered by LPS, IFN-γ and oAβ from ramified to broaden processes or spreading lamella shape (Figure 5a).

**Figure 6:**
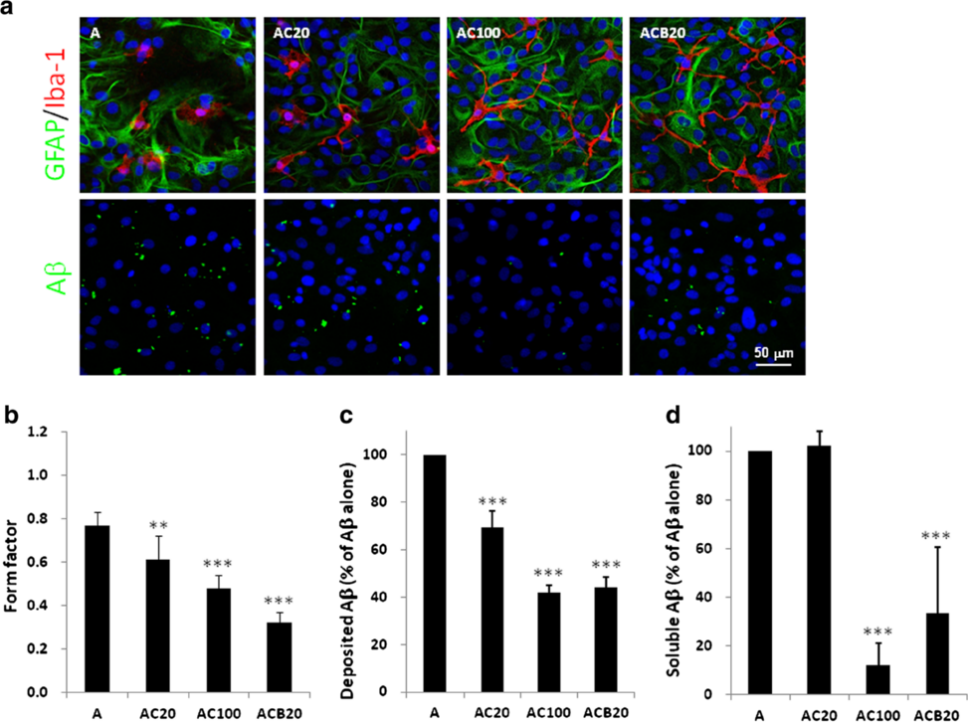
**CC-EW and CCB inhibit oAβ-induced morphological alteration of microglia and reduce the Aβ level.** Glia were treated with vehicle (A), 20 μg/ml CC-EW (AC20), 100 μg/ml CC-EW (AC100), or 20 μM CCB (ACB20) for 2 h, and then were treated with 5 μM oAβ for 24 h. **(a)** Cells were fixed and subjected to immuno-staining by anti-Iba1 antibody (red), anti-GFAP antibody and anti-Aβ antibody (green). Nuclei were stained using Hoechst 33258 (blue). Results were repeated for three times, and represent photograph are shown. **(b)** Microglial form factor were calculated as describe in the Method section. Results are means ± S.D. from three independent experiments and total nine cells of each group were analyzed. **(c)** The number of Aβ-immunoreactive deposit per field (250 μm × 250 μm) of view was calculated using Metamorph software, and presented in percentage of the cells treated with Aβ alone. **(d)** The conditioned medium was collected and subjected to Aβ1-42 ELISA assay. The data is presented in percentage of the cells treated with Aβ alone. Results are means ± S.D. from three independent experiments. Significant differences between oAβ group and other treated group are indicated by **, p < 0.01; ***, p < 0.001.

CC-EW and CCB inhibited the morphological alteration induced by oAβ and the IC_50_ is 30.00 ± 3.65 μg/ml and 10.02 ± 2.11 μM, respectively (Figure 6a, 6b; Table 1). On the other hand, CC-EW and CCB did not significantly affect the morphology of astrocytes (Figure 6a).

### The effect of CC-EW and CCB on deposit and soluble oAβ in mixed glial culture

The effect of CC-EW and CCB on recovering morphological alteration may be attributable to that CC-EW and CCB promote oAβ clearance by microglia and/or astrocytes. This hypothesis was verified by detecting the Aβ deposition on cells and the remaining soluble Aβ in the conditioned medium. Therefore, the immunostaining of the deposit Aβ on cells was performed and the soluble Aβ was determined by ELISA assay. The results showed that the Aβ deposition was significantly reduced by CC-EW and CCB, and IC_50_ is 32.67 ± 2.69 μg/ml and 17.82 ± 1.78 μM, respectively (Figure 6c, Table 1). The remained soluble Aβ in the conditioned medium was also significantly reduced by CC-EW and CCB (Figure 6d).

## Discussion

The pro-inflammatory activation of microglia is one of the major events in the pathology of AD. However, the activation of microglia has been suggested to help clear Aβ by either promoting phagocytosis or enzymatic degradation [13-15] and to secret neurotrophic factors [12]. In another word, microglia could exert beneficial and detrimental effects on AD therapy [8,10]. Accordingly, some studies have been conducted to develop herbal medicines to switch microglial phenotype from detrimental to beneficial on neuronal survival after the insult of Aβ [21], which may be further developed into therapeutic medicine for AD.

In the present study, we demonstrated that the components of *C. cochinchinesis* (CC-EW and its six pure isolated compounds) attenuates the fAβ-mediated pro-inflammatory activation of the IFN-γ-primed BV-2 cells, prevents oAβ-mediated activation that was defined by the morphological alteration, and promotes Aβ clearance by glia-dependent phagocytosis and/or proteolysis in the primary mixed glial culture. It has been indicated previously that oAβ possesses the ability to stimulate nitric oxide production from the activated BV-2 cells [22] or primary microglia [23]. Furthermore, it has been suggested that oAβ and fAβ stimulate differential activation on primary microglia [24], since oAβ is a stronger M1-inductor than fAβ [25]. Alternatively, the functional genomics approach has indicated that Aβ may activate BV-2 cells to induce several gene expressions that did not include iNOS [26]. In our study, fAβ or oAβ alone failed to activate BV-2 cells to produce nitric oxide. The potential reasons for this could be attributed to fAβ may only activate the IFN-γ-primed microglia [8,27]. In our present results, fAβ, but not oAβ, enhanced the nitric oxide production through the promotion of iNOS expression in the IFN-γ-primed BV-2 cells. The failure of oAβ to activate IFN-γ-primed BV-2 cells may be due to oAβ as more accessible than fAβ to microglial phagocytosis [28] and/or the enzymatic degradation mediated by IDE by BV-2 cells [29].

CCA (1,3,5-trihydroxy-4-prenylxanthone), CCB (1,3,7-trihydroxy-4-prenylxanthone), and other four flavonoids were found to inhibit nitric oxide production. In another recent study, 1,3,5-trihydroxy-4-prenylxanthone has been showed to repress LPS-induced iNOS expression in RAW264.7 macrophage via impeding posttranslational modification of interleukine-1 receptor-associated protein kinase 1 (IRAK-1) [2]. LPS selectively bind to toll-like receptors 4, which have been suggested to be required for fAβ to stimulate microglial activation. Therefore, CCA and CCB may also repress fAβ-induced iNOS expression in the IFN-γ primed BV-2 cells via impeding posttranslational modification of IRAK-1. Moreover, luteolin has been suggested to trigger global changes in the microglial transcriptome leading to a unique anti-inflammatory and neuroprotective phenotype in BV-2 cells [30]. In our study, four flavonoids inhibited nitric oxide production, which may be accomplished via a similar pathway.

Microglia in the healthy mature CNS has a ramified morphology with a small soma and some fine cellular processes [3]. This typical appearance has been associated with microglial “resting” state. Upon different stimuli, the ramified resting microglia can be transferred into distinct activation phenotypes including the ramified alternative activation phenotype and the amoeboid classical activation phenotype [3]. Since the morphological change of microglia has been shown to link the functional phenotypes, the ramified morphology was maintained [5]. Nevertheless, *in vitro* studies on ramified microglia have been hampered because the morphology of the isolated microglia was amoeboid rather than ramified. Several studies have shown that astrocytes play a significant role in the differentiation and transformation of microglia. The amoeboid microglia has been shown to become ramified when cultured on monolayers of astrocytes [31]. Therefore, the ramified microglia in the mixed glial culture was employed in the present study. Based on our results, LPS, IFN-γ, and oAβ significantly activate the microglia in the mixed glial culture and CC-EW; and CCB prevented the oAβ-mediated morphological transformation from ramified to amoeboid. Interestingly, LPS, IFN-γ, and oAβ induced an un-uniformed morphological transformation of astrocytes, which is not comparable with the reactive astrocytes *in vivo*.

Previous studies have indicated that modulation of microglial receptor may enhance microglial Aβ phagocytosis while suppressing bystander damage to neurons from Aβ activated microglia [32]. Therefore, we hypothesize that aggregated Aβ stimulates the beneficial microglial phagocytic response and neurotoxic glial innate immune response. As the microglial phagocytosis fails to remove aggregated Aβ, the prolonged innate immune response becomes neurotoxic. This model of AD pathogenesis implies that stimulated microglia enhances Aβ clearance and suppressed microglial activation to prevent bystander damage to neurons will be a potential therapeutic.

## Conclusions

CC-EW and CCB effectively reduced the level of nitric oxide and iNOS stimulated by IFN-γ combined with fAβ. On the other hand, oAβ alone effectively activated microglia in the mixed glial culture that defined by the morphological change from ramified to amoeboid. CC-EW and CCB effectively recovered the Aβ-mediated morphological change of microglia and decreased the level of deposited and soluble Aβ by promoting its clearance. Taken together, CC-EW and CCB have potential for therapeutic intervention of AD.

## Abbreviations

Aβ: Amyloid β protein; AD: Alzheimer’s disease; BSA: Bovine serum albumin; CC-EW: Ethanol/water extracts of *Cudrania cochinchinensis*; CNS: Central nervous system; DMEM: Dulbecco’s Modified Eagle’s Medium; fAβ: Fibrillary Aβ; IDE: Insulin degrading enzyme; FBS: Fetal bovine serum; IFN-γ: Interferon-γ; iNOS: Inducible nitric oxide synthase; IRAK-1: Interleukin-1 receptor-associated protein kinase 1; LPS: Lipopolysaccharide; M-CSF: Macrophage colony-stimulating factor; oAβ: Oligomeric Aβ.

## Competing interests

The authors declare that they have no competing interests.

## Authors’ contributions

CJ carried out the cell line culture and participated in the drafted the manuscript. CC carried out the herbal extraction, purification, and identification. HJ participated in the drafted the manuscript. FY carried out the primary mixed-glial culture and performed the statistical analysis. MF carried out the amyloid preparation and identification. YJ conceived of the study, participated in its design and coordination, and helped to draft the manuscript. All authors read and approved the final manuscript.
